# Analysis of *IL-33* gene polymorphism (rs11792633 C/T) and risk of schizophrenia

**Published:** 2016-03

**Authors:** Dor Mohammad Kordi-Tamandani, Ahmad Reza Bahrami, Raziye Sabbaghi-Ghale-no, Hanieh Soleimani, Tayebe Baranzehi

**Affiliations:** 1 Department of Biology, University of Sistan and Baluchestan, Zahedan, Iran; 2 Department of Biology, Ferdowssi University of Mashhad, Mashhad, Iran

**Keywords:** *IL-33*; Polymorphism; Schizophrenia

## Abstract

Recently, inflammation has been found to be a significant factor in the development of Schizophrenia (SCZ). The aim of the present research was to investigate whether interleukin-33 (*IL-33*, OMIM: 608678) gene polymorphism (rs11792633, C/T) is associated with the development of SCZ or not. DNA was isolated from the serum of 70 patients with SCZ and 70 healthy controls. The PCR based method was used for detection of the *IL-33* polymorphism. The CT (OR=0.05, 95% CI: 0.003-0.057, P<0.001) and TT (OR=0.12, 95% CI: 0.028-0.46, P<0.001) genotypes significantly decreased the risk of SCZ. Our present findings indicate that the *IL-33* polymorphism associated with the risk of SCZ.

## INTRODUCTION

Schizophrenia (SCZ) is a genetically complex mental disorder, which may affect up to 1% of the world’s population. There is some evidence suggesting that cytokine production during chronic activation of the immune system ,which is observed in SCZ, could modulate prodromes, the active residual phases of this disease, and influence response to treatment [1, 2]. Interleukin-33 (*IL-33*, OMIM: 608678) is a 30kD protein with a length of 270 amino acids in two domains, a helix-turn-helix domain and an IL-1-like domain for binding to and activation of the ST2 receptors [3, 4]. *IL-33* can act as either a pro-inflammatory or an anti-inflammatory cytokine [4-6]. Recently it has been described as a member of the IL-1 family. This cytokine has a dual-function with nuclear and extracellular effects [4]. The critical role of this cytokine in allergic inflammatory diseases such as rhinitis is well known [6]. *IL-33* is a ligand of the IL-1R family member ST2 (also called ST2L, T1, Der-4), which makes part of the Toll-like receptor (TLR)/IL1R super family [7]. The producers of *IL-33* are different immune cells such as macrophages, dendritic cells and mast cells (in these cells, IL-33 induces the secretion of chemokines and cytokines such as IL-6, IL-8, and IL-13). In addition, some of the non-immune cells including endothelial, epithelial, smooth muscle cells and fibroblasts are producers of this cytokine [8]. Basal *IL-33* mRNA levels are extremely high in the brain and spinal cord. Furthermore, the expression of *IL-33* in glial and astrocyte cultures is increased by Toll-like receptor ligands. Treatment with *IL-33* induces the proliferation of microglia and enhances the production of pro-inflammatory cytokines such as *IL-1b* and *TNFα* as well as the anti-inflammatory cytokine *IL-10*. It also enhances chemokines, along with nitric oxide production and phagocytosis by microglia [9]. A better understanding of the molecular events that occur during inflammation within the central nervous system could lead to the development of novel therapeutic strategies to treat disorders associated with activation of inflammatory pathways in the brain. The aim of the present study was to determine the association of *IL-33* gene polymorphism *(*rs11792633, C/T*)* with the development of SCZ.

## MATERIALS AND METHODS

This case-control study was approved by the Ethics Committee of Azadi Hospital, Tehran, Iran and conducted in 2010–2011. The subjects consisted of a healthy control group (n=70), who were free from any signs of neuropsychiatric diseases, and a patient group diagnosed with SCZ (n=70). Serum samples for DNA extraction were collected in tubes. For DNA extraction, a Cinnapure DNA purification kit was used. Polymorphism was identified by PCR using a Tetra Amplification Refractory Mutation System (Tetra ARMS–PCR). Sequences of primers used are listed in Table 1. 

**Table 1:** Primers sequences and annealing temperatures for rs11792633 polymorphism

Primer type	Orientation	Primer Sequence(5ʹ3ʹ)	Tm (ᵒC)
Outer primers	Forward	TGCTTGTCCTACTAGATGCTAGCCCCCACA	72
	Reverse	GCATGATTTTGGTGGAAACATTCAAACCA	72
Inner primers	Forward	CCCAGAGTCCACACTCAGTATTAGGCAGG	72
	Reverse	TAGTCAGCATCACATGGGAACGTGATCGA	73

Statistical analysis was conducted using SPSS statistical software, version 16 (SPSS, Chicago, IL) and Epical version3.2. Categorical data were analyzed by Pearson’s *x*^2^ test.

## RESULTS AND DISCUSSION

The main characteristics of the populations are described in Table 2. There were differences between cases and controls for smoking habit, educational levels, and marital status. 

As shown in Table 3, CT (OR=0.05, 95% CI: 0.003-0.057, P<0.001) and TT (OR=0.12, 95% CI: 0.02-0.46, P<0.001) genotypes significantly decreased the risk of SCZ. Studies have revealed that one of the abnormalities found in SCZ includes changes in the immunological system [10].

**Table 2:** The socio-demographic characteristics of the case and control groups

**Variable**	**Cases**	**Controls**	**P**
**Age**	47.5+10.8	46.7+11.7	>0.05
**Age of onset**	20.7+8.7	-	-
			
**Sex**			
**Females**	30	44	>0.05
**Males**	40	26	
			
**Smoking status**			
**Non smokers**	-	45	<0.001
**Smokers**	70	25	
			
**Educational level**			
**Illiteracy**	3	-	
**Primary School**	8	1	
**Guidance**	16	5	<0.001
**High school**	54	28	
			
**Marital status**			
**Single**	50	29	
**Married**	17	41	<0.001
**Divorced**	16	6	

**Table 3:** Association between polymorphism of *IL-33* rs11792633 C/T) and risk of schizophrenia

**Genotypes**	**Cases (%)**	**Controls (%)**	**OR**	**95% CI**	**P**
CC	41 (58.5)	4 (5.7)	1.0	-	-
CT	7 (10.0)	49 (70.0)	0.05	0.003-0.057	<0.001
TT	22 (31.4)	17 (24.2)	0.12	0.028-0.46	<0.001
CT+TT	29	66	0.04	0.01-0.13	<0.001

We hypothesized that patients with SCZ may demonstrate a different distribution of allele frequencies in the *IL-33* gene from controls. In this study, we have reported the significant association of rs11792633 SNP in the *IL-33* and risk of SCZ in an Iranian population. *IL-33* may also play a protective role in the development and progression of atherosclerosis [12]. Recently, functional studies have shown that the minor alleles, rs1157505, rs11792633, and rs7044343 within *IL-33* were associated with a lower degree of cerebral amyloid angiopathy in the brains of non- apolipoprotein E, and IL-33 expression was reduced in the brains' of patients with Alzheimer’s disease. Cellular models have suggested that this association may be due to the ability of *IL-33* to down-regulate A- peptide secretion [11]. A major biological action of this cytokine was recently described as a crucial intracellular nuclear factor with transcriptional regulatory properties associated with Crohn’s disease [12]. All in all, our results showed that there is an association between *IL-33* gene polymorphisms and risk of Schizophrenia in our population. However, we suggest that the results of this study be confirmed in further investigations using larger sample size in different populations across the world. 

## Notes

**Acknowledgements: **We would like to thank the Department of Biology, University of Sistan and Baluchestan, Zahedan, Iran for supporting this project financially.
